# Cold Rolling Deformation Characteristic of a Biomedical Beta Type Ti–25Nb–3Zr–2Sn–3Mo Alloy Plate and Its Influence on α Precipitated Phases and Room Temperature Mechanical Properties During Aging Treatment

**DOI:** 10.3389/fbioe.2020.598529

**Published:** 2020-10-19

**Authors:** Jun Cheng, Jinshan Li, Sen Yu, Zhaoxin Du, Xiaoyong Zhang, Wen Zhang, Jinyang Gai, Hongchuan Wang, Hongjie Song, Zhentao Yu

**Affiliations:** ^1^State Key Laboratory of Solidification Processing, Northwestern Polytechnical University, Xi’an, China; ^2^Shaanxi Key Laboratory of Biomedical Metal Materials, Northwest Institute for Non-ferrous Metal Research, Xi’an, China; ^3^School of Materials Science and Engineering, Inner Mongolia University of Technology, Hohhot, China; ^4^State Key Laboratory of Powder Metallurgy, Central South University, Changsha, China; ^5^School of Material Science and Engineering, Northeastern University, Shenyang, China; ^6^Institute of Advanced Wear and Corrosion Resistant and Functional Materials, Jinan University, Guangzhou, China

**Keywords:** Cold deformation, biomedical β-type titanium alloy, α precipitated phases, mechanical properties, texture evolution

## Abstract

The microstructure characteristics and texture evolution of a biomedical metastable beta Ti–25Nb–3Zr–2Sn–3Mo (TLM; wt%) titanium alloy plate cold rolled at various reductions were studied in this article. <110> texture was easily formed in the TLM alloy plates, and a large number of dislocation tangles were generated in the β matrix in the process of cold rolling deformation. The dislocation lines, dislocation cells, subgrain boundaries, and other crystal defects introduced during cold rolling had a great impact on the morphological characteristics and volume fraction of precipitated phases during aging. These typical crystal defects could be considered as the major triggers of the formation of second phases, and they could also shorten the time of β→α phase transformation. α precipitated phases, with a size range of 150–500 nm, were formed within the β matrix in the cold deformed 34% in conjunction with the aging specimen, resulting in the relatively high tensile strength of 931 MPa and the acceptable elongation of 6.9%. When the TLM alloy plate was cold rolled at a reduction of 60% in conjunction with aging, the maximum value of ultimate strength (1,005 MPa) was achieved, but the elongation value was relatively low owing to the formation of α precipitated phases with a large size around the subgrain boundaries. In this paper, the influence of crystal defects and subgrain boundaries on the morphology characteristics and volume fraction of α precipitated phases and mechanical properties will be discussed in detail.

## Introduction

Compared with other noble metals (Au, Ag), medical stainless steel (316L), magnesium alloys (Mg–4Y–3RE), and Co–Cr–Mo alloys (Co–28Cr–6Mo), Ti, and its alloys possess more outstanding comprehensive performances in the metallic implant material family (Rack and Qazi, 2006; Niinomi et al., 2012; Zhang and Chen, 2019; Liu et al., 2020). Both large application potential and commercial values exist in hard tissue reconstruction and replacement due to their attractive characteristics, including low modulus, excellent mechanical properties, as well as superior biofunctions and biocompatibility (Niinomi, 2002; Geetha et al., 2009; Niinomi and Nakai, 2011; Zhang et al., 2020). Hence, Ti and Ti alloys are extensively applied in the processing and manufacturing of dental implants, bone plates, spinal internal fixation devices, orthodontic wires, intramedullary nails, as well as other orthopedic repair devices (Taddei et al., 2004; Li Y. et al., 2014; Duraccio et al., 2015). Currently, commercial Ti64 and Ti–13Nb–13Zr are mainly applied to produce devices for joint prosthesis. However, commercial Ti64 contains toxic elements, such as Al and V, which may cause Alzheimer’s disease after long-term implantation in the patient’s body (Gepreel and Niinomi, 2013; Abdelrhman et al., 2019). The biomedical β Ti alloy is predominantly composed of biocompatible components, including Nb, Zr, Sn, Mo, Ta, and Fe (Wang L. et al., 2010; Malek et al., 2012). In general, the strength level in biomechanical properties is crucial for the long-term service and safety of metallic implant materials and their devices (Goriainov et al., 2014). Notably, β Ti alloys can achieve a comprehensive matching degree with high strength, good biocompatibility, and acceptable ductility through optimized processing (Niinomi, 2008; Ramezannejad et al., 2019). In addition, based on the effect of precipitation strengthening, the strength level of β-type Ti alloy can be significantly improved after solution treatment followed by rapid cooling and aging (Banerjee et al., 2004; Chen et al., 2014; Xu et al., 2019b). Up to now, several typical metastable β Ti alloys, for instance, Ti–Mo, Ti–Nb, Ti–V, and Ti–Ta-based alloys, have received considerable attention due to their controllable microstructures and performances (Mantani and Tajima, 2006; Sun et al., 2010; Min et al., 2012). A detailed investigation of the influences of heat treatment on microstructural evolution, phase transitions, mechanical properties, and deformation mechanisms has been carried out for metastable β Ti alloys. Moreover, for β-type Ti alloys, a suitable heat treatment process is beneficial to produce fine, uniform, and dispersed α precipitates in the β matrix, thereby improving the mechanical properties of β-type Ti alloys (Jaworski and Ankem, 2005; Qazi et al., 2005). It is significant to note that the density, distribution, and length-to-diameter ratio for secondary phases are considered to be the primary factors affecting the strength level of β-type Ti alloys. Previous studies indicated that the improvement of strength for Ti alloys can be achieved using the composite processing of cold rolling followed by aging at a certain condition (Malinov et al., 2003). A large number of dislocation tangles/proliferation, nucleation sites, and sub-boundaries (interfaces) would be introduced into the deformed microstructure of β-type Ti alloy during the plastic deformation. The presence of defects is considered to be beneficial for the refinement and uniform distribution of precipitation phases. Meanwhile, grain refinement is apparent (Xu et al., 2016a). In general, the crystal defects introduced during plastic deformation are mainly composed of dislocations, twins, and interfaces, etc., which can facilitate the β→α phase transition (Wang et al., 2009; Karthikeyan et al., 2010). The primary reason is that the presence of defects is able to reduce the driving force and barrier required to the occurrence of the phase transition to some extent.

Until now, a large number of groups are persistently focusing on the microstructural controlling, the mechanical property optimization, and the relationship between them, resulting in a lot of representative studies (Ivasishin et al., 2005; Xu et al., 2016b). Li T. et al. (2014) prepared β-type Ti–15Nb–2Mo–2Zr–1Sn alloy with ultrafine grains using hot or cold working followed by aging. Finer precipitates were prone to be generated at the elongated grain boundaries or subgrain boundaries, resulting in a significant improvement of the room temperature strength level. Karthikeyan et al. (2010) studied the influence of cooling rate on texture feature and variant selection in phase transition for Ti–5Ta–1.8Nb alloy during aging. They found that the effect of cold rolling on the microstructure and phase transition in the subsequent annealing has a significant inheritability. Song et al. (2010) discussed the influence of predeformation in conjunction with aging on the microstructural features and room-temperature mechanical properties for β-type Ti–10Mo–8V–1Fe–3.5Al alloy. The results showed that the alloy is only subjected to predeformation and single aging. Although higher strength of the alloy could be achieved at room temperature, the ductility would be very poor. The final dislocation density could be reduced, and the plasticity level could be further enhanced after a lower-temperature pre-aging followed by a higher-temperature secondary aging treatment. Guo et al. (Qiang et al., 2011) revealed the precipitation progress and mechanism of the secondary phase and its effect on the mechanical properties of TB5 (nominal composition: Ti–15V–3Cr–3Sn–3Al) alloy after severe plastic deformation. They found that finer precipitates are generated within the region of severe deformation and evenly distributed within the β grains, while in other transition regions, the newly precipitated phases are thick lamellar or in a shuttle-like shape with relatively large size. Therefore, in the processing of the semifinished products made of β-type Ti alloys, the finer and uniformly dispersed precipitates can be obtained using a composite preparation method of cold deformation in conjunction with aging treatment, leading to an obvious improvement in strength level. However, the strengthening mechanism of biomedical β-type Ti-Nb-Zr-Sn-Mo-based Ti alloys subjected to cold deformation in conjunction with aging has not been definitely investigated and proposed until now. Although biomedical β-type Ti alloys have superior cold workability compared with α- and (α + β)-type alloys, their plastic deformation behavior is extraordinarily complicated. The deformation mechanism is not only related to phase stability but also to the reduction of deformation (Wang et al., 2015; Hafeez et al., 2020). Mechanical properties of β type Ti alloy are associated with multiple deformation modes including dislocation slip, mechanical twinning, stress-induced martensitic transformation, kinking deformation, and dislocation-free plastic deformation, etc. (Xiang et al., 2020; Zheng et al., 2020). Hence, the investigation on cold deformation behavior of β-type Ti alloys has extremely important practical significance for the development of biofunctional Ti alloys with an excellent matching degree of lower modulus and higher strength.

Ti–25Nb–3Zr–2Sn–3Mo (TLM) alloy, a type of metastable β-type titanium alloy, is independently developed for surgical implants by Shaanxi Key Laboratory of Biomedical Metal Materials (Kent et al., 2010; Yu et al., 2011, 2014). The α precipitates are generated in β grains and along with grain boundaries after solution treatment followed by aging, leading to an obvious increase in the strength level of the alloy (Salib et al., 2013). Malinov et al. (2003) revealed the phase transformation mechanism of β-21s Ti alloy under constant temperature conditions at a temperature ranging from 400°C to 750°C with the assistance of experimental research and computer simulation methods. Meanwhile, the corresponding thermodynamic, and kinetic models were established, respectively. Xu et al. (2015) investigated the effect of various heat treatments on phase transition mechanism, microstructural features, and room temperature mechanical properties for the TB8 alloy. They found that the location and morphological features of precipitates are closely related to the aging temperature and time. In addition, finer and more uniform secondary phases can be obtained by adopting an appropriate solution treatment followed by aging, leading to a significant increase in the strength level. Meanwhile, Xu et al. (2016b) investigated the microstructural features and phase transition mechanism in the two-stage aging process. They found that the isothermal omega transition phases precipitated in a single aging process could provide more nucleation sites for α phases, resulting in an apparent refinement of α phases. Kent et al. (2011) investigated the microstructural features and phase transition behavior of Ti–25Nb–3Zr–2Sn–3Mo alloy using accumulative roll bonding (ARB) processing technology. The ultrafine-grained alloy was prepared, and α precipitates with nanosize were generated along with the grain boundaries, leading to the increment in the strength level. In summary, for the biomedical metastable β-type Ti alloy, the microstructure morphology and texture component would simultaneously change during cold deformation (Ma et al., 2018; Vajpai et al., 2018; Ozan et al., 2019). This will induce the presence of preferred orientation and the increase in the dislocation density, resulting in the changing in the nucleation and growth rates of precipitates during aging. In the meanwhile, the driving force and barrier would be reduced when the phase transition of β→α occurs.

This work will mainly focus on the microstructure evolution and phase transition of TLM alloy plates after cold rolling in conjunction with aging treatment. The effects of cold deformation-induced dislocations and subgrain boundaries on the formation mechanism of precipitates and mechanical properties are also discussed. This work will provide the theoretical guidance and technology optimization for the precise control of microstructures and properties for high-performance biomedical β-type Ti alloys, which has important scientific significance and engineering value.

## Experimental Materials and Methods

**FIGURE 1:**
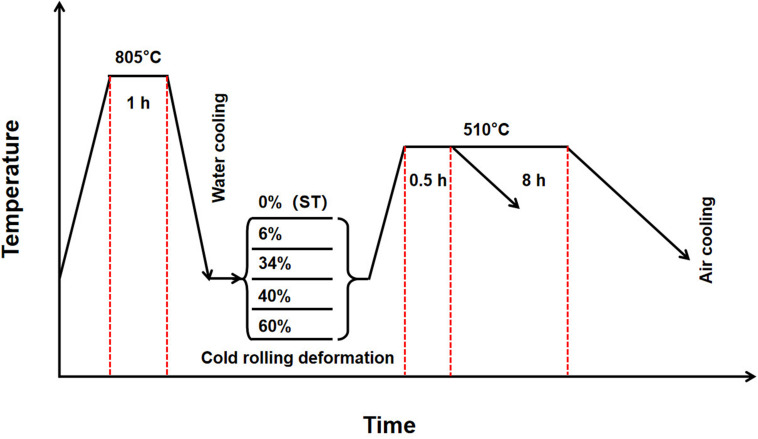
Schematic illustration of cold rolling and heat treatment routes for the Ti–25Nb–3Zr–2Sn–3Mo (TLM) alloy plates used in this work.

The TLM (Ti–25Nb–3Zr–2Sn–3Mo, wt%) alloy ingot was produced by VAR (vacuum arc remelting) three times. The sponge titanium (grade 0, 3–12.7 mm), sponge zirconium (industrial grade, Zr-1), Nb–47Ti, Ti–80Sn, and Ti—2Mo binary master alloys (chips) were used as raw materials. Consumable electrode blocks were prepared using a 1,000-ton four-column hydraulic press. Both the alloy ingots and master alloys were fabricated by NIN BRC SKLBMM (Northwest Institute for Non-ferrous Metal Research, Biomaterial Research Center, Shaanxi Key Laboratory of Biomedical Metal Materials, Xi’an, China). Clean turning scraps and bulk samples for testing were machined from ingot at two various positions. The surface of the machined sample is smooth and free of burrs. The chemical compositions of TLM alloy were examined using the inductively coupled plasma optical emission spectroscopy (ICPOES, for the metallic main elements including Mo, Zr, Sn, Fe, and Nb) and infrared absorption method (IR absorption, for the interstitial elements including N, O, H, and C). The chemical compositions of TLM alloy ingot at two different positions are listed in Table 1. The ingot with a dimension of Φ160 mm × 500 mm was multistep hot forged in order to break down the coarse grains after homogenization treatment at 1,150°C for 8 h. The TLM alloy billets were hot rolled at 750°C into plates with 5 mm in thickness using 2,000 tons of reversing rolling plate equipment. First, the hot-rolled plate billet was leveled and straightened by the automatic plate-leveling machine. Subsequently, the oxide layer of the plate was efficiently removed using sandblasting and mechanical polishing. The surface of the alloy plate was polished in the end. Then the alloy plates were solution treated (ST) at 805°C for 1 h followed by water cooling and cold rolled with reductions of 6, 34, 40, and 60% in thickness by 650-type rolling plate equipment. For the cold-rolled specimens, aging treatments were performed at 510°C for 8 h in the muffle furnace. Both the oxide layer and the micrometallurgical defects were removed by NC machines. The schematic illustration of cold rolling and heat treatment routes for the TLM alloy plates in this work is shown in Figure 1.

**TABLE 1:** Chemical compositions of TLM (Ti–25Nb–3Zr–2Sn–3Mo, wt%) alloy ingot at two different positions.

Position	Ti	Nb	Zr	Sn	Mo	Fe	C	N	O	H
Upper	Bal.	25.5	3.05	2.05	3.05	0.02	0.01	0.003	0.049	0.0006
Bottom	Bal.	25.8	2.96	2.03	3.08	0.02	0.02	0.003	0.044	0.0006

**FIGURE 2:**
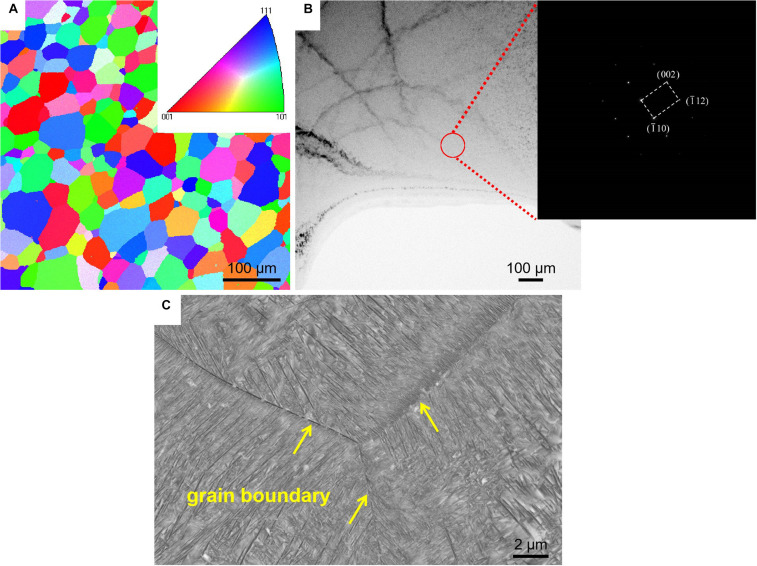
Microstructures of the TLM alloy plate. **(A)** Electron backscatter diffraction (EBSD) map of the sample solution treated at 805°C for 1 h. **(B)** Transmission electron microscopy (TEM) image for the solution-treated sample. **(C)** Scanning electron microscopy (Ivasishin et al.) image for the solution-treated (805°C/1 h, WQ) followed by aging-treated (510°C/8 h, AC) alloy plate (WQ, water quenching; AC, air cooling).

Figure 2 shows microstructures of the TLM alloy plate ST at 805°C for 1 h followed by water cooling and aging-treated at 510°C for 8 h after solution treatment. The inverse pole figure (IPF) map and transmission electron microscope (TEM) images for the ST TLM alloy plate indicate that the microstructures are constituted by β phase, as presented in Figures 2A,B. The α precipitates generated during one step aging after solution treatment exhibit a needle shape within the β matrix, as shown in Figure 2C.

The uniaxial tensile test was carried out using an INSTRON 598X (maximum load: 250 kN) machine at a constant strain rate of 5 × 10^–3^ s^–1^ at room temperature. Tensile samples were machined from TLM alloy plates in compliance with ASTM E8/E8M-13a (width: 6.0 ± 0.1 mm, gage length: 25 ± 0.1 mm). The optical microstructures (OMs) were examined by a LEICA microscope. Metallographic samples were etched using Kroll’s reagent (10 vol% HF + 10 vol% HNO_3_ + 80 vol% H_2_O). X-ray diffraction (XRD) analysis was performed by BRUKER D8 ADVANCE machine equipped with copper K_α_ radiation. The acceleration voltage and operating current were set at 40 kV and 40 mA, respectively. The XRD was conducted at 2 theta angle of 20–90°, a step size of 0.02°, and a scanning speed of 6°/min. The texture analysis was carried out using high-resolution XRD (XPert Pro MRD). The fracture morphology and microstructure morphology were observed and analyzed by a FEI Quanta 650F SEM. electron backscattered diffraction (EBSD) characterization was conducted by AZtech (Oxford Instrument, HKL Channel 5 data analysis software) with an accelerating voltage of 20 kV. The microstructural morphology of high magnification was observed using FEI Tecnai G2 F20 TEM operating at 200 kV. TEM specimens were mechanically ground to a thickness of approximately 40 μm using *SiC* abrasive paper (400, 600, 800, 1,000, 1,200, 1,500, and 2,000 grit). The ion milling was performed for the preparation and observation of the TEM samples.

## Results and Discussion

### Cold Rolling and Texture Analysis

In the process of plastic deformation of metal materials, both the dislocation density and residual stress are extremely high in the area with a large degree of deformation, which leads to a significant reduction in the calibration rate of EBSD. The quality of the obtained images is poor, and the grain shape and crystallographic orientation cannot be achieved. On the contrary, the calibration rate is higher, and the image quality is relatively better in some areas with a smaller degree of deformation. Furthermore, the calibration rate is also relatively low due to the high density of defects or dislocations at grain boundaries. In the early stage of the investigation, it was found that higher residual stress would significantly affect the calibration rate (less than 50%) in cold deformed samples using EBSD technique, resulting in blurry Kikuchi patterns. Compared with the EBSD technique, the sample preparation of XRD is simple. The specimen is irradiated with characteristic X-rays, and the texture analysis is carried out according to the difference in the diffraction intensity in different characteristic directions. The evaluation of macroscopic properties for crystalline materials is unique. Therefore, the XRD technique can effectively make up for the shortcomings of a lower calibration rate in cold deformed metallic samples. Meanwhile, this technique is more suitable for the investigation of texture evolution and crystallographic orientation relationship of titanium and its alloys during cold deformation, such as cold rolling and cold drawing.

**FIGURE 3:**
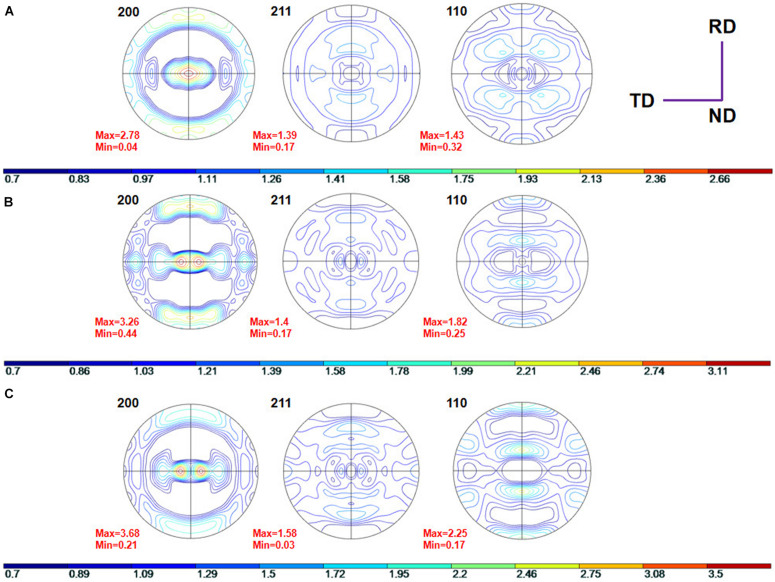
The {200}, {211}, and {110} pole figures (PF) for the TLM alloy plate after cold rolling at various reductions: **(A)** 6%, **(B)** 34%, and **(C)** 60%.

Figure 3 shows the pole figures (PF) of TLM alloy plates cold deformed with 6, 34, and 60% reductions in thickness measured by XRD. It can be seen from Figure 3A that when the TLM alloy plate is cold rolled with a 6% reduction, the β grains present a kind of relatively random orientation distribution characteristic, and only a small part of the β grains is rotated during cold rolling. The main reason for the rotation of the β grains is that dislocation tangles are dominant for the TLM alloy plate during cold rolling at a relatively small reduction. The elongated β grains can be observed in the cold-rolled alloy plates, and few secondary phases or recrystallized grains were generated. In addition, as seen from the PF in Figures 3B,C, with the increased degree of cold rolling deformation, the <110> texture gradually appears and increases. As shown in Figure 3B, when the TLM alloy plate is cold deformed with 34% reduction, the intensity of both <200 > and <110> textures increase. Meanwhile, when the reduction is raised to 60%, the TLM alloy plate has an obvious <110> texture. It can be reasonably inferred that the β grains tend to rotate to the <110> direction instead of <211 > during cold rolling. Generally, for β-type titanium alloys with a body-centered cubic (BCC) structure, with the increase in the cold reduction, the <110> texture is easier to be presented due to the tightness of atomic arrangements and slip systems on the close-packed plane during the plastic deformation (tension, compression, and rolling) at room temperature.

### Cold Rolling, Dislocation Distribution, and Substructure

**FIGURE 4:**
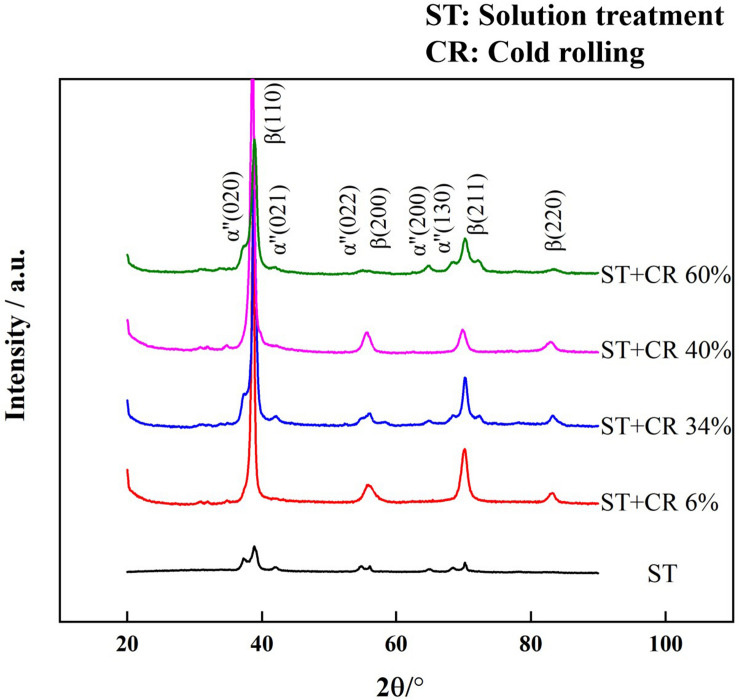
X-ray diffraction (XRD) spectra for the TLM alloy plate after cold rolling with reductions of 0% (solution treatment), 6, 34, 40, and 60%.

X-ray diffraction patterns of the TLM specimen ST at 805°C for 1 h followed by water quenching and cold rolling at various reductions are indicated in Figure 4. Diffraction peaks of orthorhombic a “phases are obviously detected in the alloy plate ST at 805°C for 1 h. Meanwhile, a few stress-induced martensite a” phases gradually appear after cold rolling with various reductions at room temperature. It can be inferred that the phase transition of stress-induced martensite from β phase to a “is promoted after cold deformation. In addition, the intensity and quantity of diffraction peaks for a” phase gradually increases with the increase in the degree of reduction owing to the martensite phase transition that is prone to be promoted after cold rolling. It can be observed that (110)_β_, (200)_β_, (211)_β_, and (220)_β_ diffraction peaks are detected in all specimens. Moreover, with the increase in the cold reduction, the intensity of (200)_β_ diffraction peak first decreases up to the reduction of 34% and subsequently increases up to the reduction of 40% and then decreases again after 60% reduction. The ratios of β (200)/β (110) are calculated to be 0.209, 0.037, 0.029, 0.055, and 0.018, respectively. This finding demonstrates that the texture varies with the reduction in the cold deformation; the textures of cold-deformed TLM alloy plate gradually transform into the <110> orientation during cold working. Generally, the full width half maximum (FWHM) in XRD patterns of deformed metals is used to analyze the lattice distortion owing to the dislocation movement, deformation twins, and kinking, etc. The FWHM value of β (110) diffraction peak obtained using Jade 6.0 are 0.494, 0.527, 0.597, and 0.697 for the samples cold deformed at 0 (solution-treated), 6, 34, 40, and 60% rolling reduction, respectively. It is clearly seen that with increasing cold-deformed reduction, the values of FWHM of β (110) diffraction peak show a rising tendency. Furthermore, the change in the FWHM of β (110) diffraction peak in various cold-deformed samples illustrates that dislocation tangles and substructures are the predominant deformation mechanisms in severe plastic deformation samples. The cold-rolled TLM alloy plate with a large number of dislocation tangles tends to inhibit the generation of dislocation cells and subgrains, especially in BCC alloys. A lot of deformation bands are generated within the deformed microstructures for the coordination deformation. Therefore, the deformed grains and newly formed substructures, such as subgrains, subgrain boundaries, and deformation twins, could be found in the TLM specimens after severe plastic deformation (Hao et al., 2012; Xu et al., 2019a).

**FIGURE 5:**
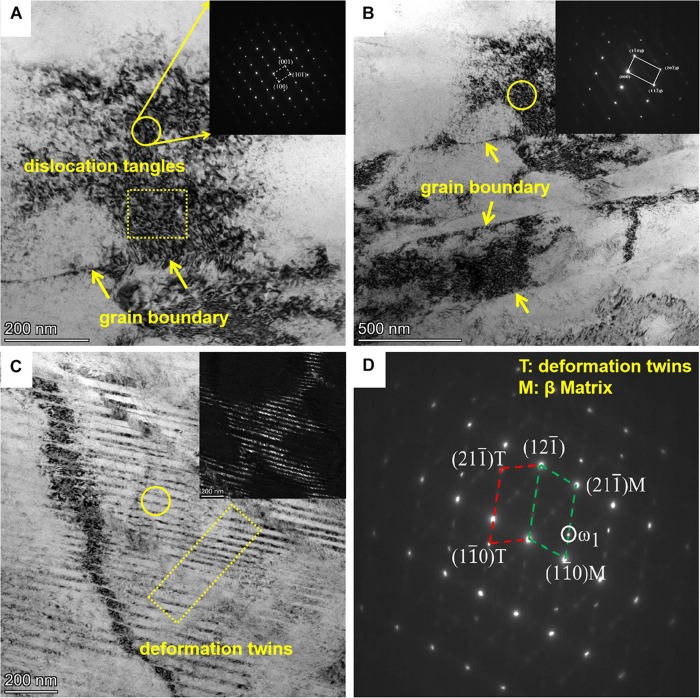
TEM analyses of the TLM alloy plates cold rolled with various reductions. **(A,B)** Bright-field (BF) micrographs and SAED patterns for the specimen cold deformed with the reduction of 34%. **(C,D)** Bright-field (BF) micrographs and SAED patterns for the specimen cold deformed at a reduction of 60%.

Transmission electron microscope micrographs for the TLM alloy plate after cold rolling at 34 and 60% reduction. Bright-field images (BF) are used to reveal the crystallographic misorientation and characteristics of cold-deformed microstructures. Dislocation density in the cold-deformed TLM specimen (Figure 5) is higher than that in the solution-annealed alloy (Figure 2B). Therefore, it could be deduced that the dislocation multiplication, mutual reactions, and movement play significant roles in the cold rolling of TLM alloy plate. It can be seen from the SAED pattern (Figure 5A) that the black bands scattered within the cold-deformed TLM alloy are not newly formed phases or other structures but the dislocation tangles generated in the process of cold rolling. The density of dislocation increases visibly after cold rolling at 34% reduction. Bright-field image taken from another region of the 34% cold-deformed TLM alloy plate is presented in Figure 5B, which displays the dislocation pile-up near the grain boundaries as well as in the grain interior. As seen from the SAED patterns (Figure 5B), phase transition and newly formed substructures are observed in the region of black and white bands. This interesting phenomenon was previously found by Takemoto in the investigation of tensile deformation behavior and cold rolling at various reductions for the titanium–molybdenum alloy (Takemoto et al., 2004). The formation of bands is attributed to the dislocation movement and tangle non-uniformity to some extent. Additionally, it can be observed that the extension and growth of bands are suppressed by the grain boundary. The bright-field (BF) image and SAED patterns for the 60% cold-deformed TLM alloy plate are displayed in Figures 5C,D. We can also see that a severe deformation area is primarily composed of dislocation tangle and 112 <111 > type deformation twins near the grain boundary. Generally, the ω phases induced by stress during cold rolling were not detected by XRD and TEM in the cold-deformed TLM alloy (Cheng et al., 2020). One of the reasons is that the contents of β stable elements, such as Nb, Zr, Sn, and Mo, are so high that the formation of ω precipitates is suppressed to a large extent. This kind of ω phase can be considered as stress-induced omega (SIO) phase transformation triggered by the recombination of solute atoms in the {112} lattice planes with the orientation of <111 > crystallographic direction families. The mechanism of dislocation motion and tangles can also be clarified by the replacement of solute atoms in {112} lattice planes in the direction of <111 > during cold deformation. Moreover, the degree of dislocation pile up improves obviously with the increment in the reductions, which may result in the generation of new boundaries for the β-type Ti alloys (Wu et al., 2014; Yang et al., 2018; Rabadia et al., 2019a, b).

### Aging Treatment, Precipitation Phases, and Mechanical Properties

**FIGURE 6:**
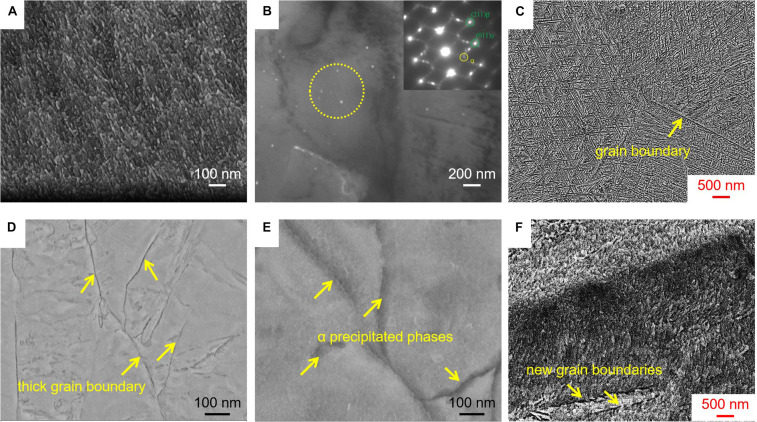
Micrographs of the TLM alloy plate cold rolled with various reductions in conjunction with aging treated at 510°C. **(A,B)** SEM image, dark-field TEM micrograph, and SAED pattern for the sample after cold rolling at a reduction of 34% in conjunction with aging treatment for 0.5 h. **(C)** SEM image for the sample after cold rolling at a reduction of 34% in conjunction with aging treatment for 8 h. **(D,E)** SEM images for the sample after cold rolling at a reduction of 60% in conjunction with aging treatment for 0.5 h. **(F)** SEM image for the sample after cold rolling at a reduction of 60% in conjunction with aging treatment for 8 h.

In general, the β→α phase transformation could be triggered in the TLM alloy during the solution treatment plus aging. Figure 6 presents the morphology (SEM and TEM) of the precipitation phases for the alloy after cold rolling at various reductions plus 510°C aging treatment. As can be seen from Figures 6C,F, compared with the size of precipitated phases in the single aging-treated sample, the precipitations formed in the TLM alloy subjected to cold rolling in conjunction with the same aging should be refined to a large extent. When the TLM alloy plate is cold rolled at a reduction of 34% in conjunction with aging treatment for 8 h, the length of precipitation phases is approximately 150–500 nm, which is much thinner than that of a single aged one. The formation of dislocation lines or dislocation cells introduced by cold rolling deformation plays a very important role in the emergence of precipitates during aging treatment. Figure 6B presents the dark-field (DF) TEM picture of the morphology for the tiny precipitations generated in the process of cold rolling at a 34% reduction followed by short-time aging treatment. As seen from the SAED pattern, the white precipitation phases should be α phase. Systematic research on α precipitation phases with nanometer dimensions of the initial sample, such as β21s, subjected to certain aging heat treatment was carried out. However, α phases were hardly observed in the β matrix using an electron microscope (Malinov et al., 2003; Banerjee and Williams, 2013). The reason for this interesting phenomenon is that the crystal defects, such as dislocation lines, sub-boundaries, phase interface, and twin boundaries, can promote the phase transition of β→α and reduce the time of subsequent aging simultaneously (Wang Y. B. et al., 2010; Guo et al., 2013). Figures 6A,B present that the length of α precipitated phases is approximately 15–90 nm in the sample cold rolled at 34% reduction followed by aging treatment for 0.5 h. After rolling, a large number of dislocations were introduced into the initial microstructure, which is favorable for the homogeneous nucleation process of β→α phase transition during aging, resulting in the refinement of α precipitations. More and more nucleation sites in the β matrix will be conductive to suppress the rapid growth of the α phase. Furthermore, a large number of newly generated interfaces were introduced into the deformed specimen. These new interfaces accelerate the precipitation of lamellar α phases, which may result in a decrease in the plasticity of aged specimens. The newly generated grain boundaries contribute to the homogeneous nucleation and dispersed precipitation of α phase in the process cold rolling at various reductions. Moreover, it can be seen from Figures 6D–F that the newly generated grain boundaries appear during cold rolling at a relatively large reduction (60%), resulting in the linear dispersion of secondary phases along with them after aging treatment.

Previous studies have shown that the dislocation density of alloy is approximately 1.0 × 10^9^ mm^–2^ after plastic deformation (Azushima et al., 2008; Cheong, 2008). During cold rolling processing, the formation of dislocations in the TLM alloy plate could provide the driving force for β→α transformation after aging, which contributes to the refinement of the α phase. Therefore, the strengthening mechanisms for the TLM alloy plate cold rolled with various reductions followed by aging could be clarified using the above theory. This kind of finer α phase can offer more phase interfaces, which will be regarded as one type of powerful dislocation obstacles, and can also help to obtain the enhancement of strength for the TLM alloy.

**FIGURE 7:**
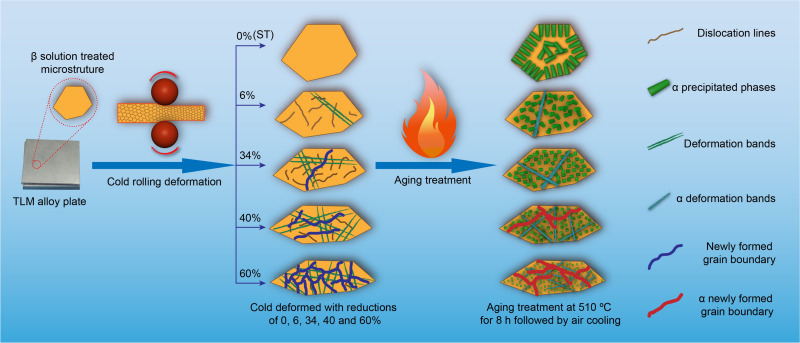
A schematic diagram of microstructural evolution and precipitating process for the TLM alloy plates cold rolled with reductions of 0, 6, 36, 40, and 60% followed by aging treatment at 510°C for 8 h.

A schematic diagram of microstructural evolution and precipitating process for the TLM alloy plates cold rolled with reductions of 0, 6, 36, 40, and 60% followed by aging treatment at 510°C for 8 h is presented in Figure 7. The morphology of the α phases shows a remarkable difference at various cold-rolling reductions. Meanwhile, a large number of slip bands and newly generated grain boundaries gradually emerge during cold deformation. To the author’s knowledge, they are considered as a typically inferior microstructural characteristic, which is prone to be changed into coarse lamellar α precipitates after following aging treatment. The dislocation slip or motion is suppressed by this microstructure coarsening, and the ductility is simultaneously weakened during tensile deformation at room temperature. In addition, when the plate is cold rolled with a reduction of 60% in conjunction with aging treatment at 510°C for 8 h, the maximum strength value (tensile strength: 1,005 MPa) is achieved under this condition.

**FIGURE 8:**
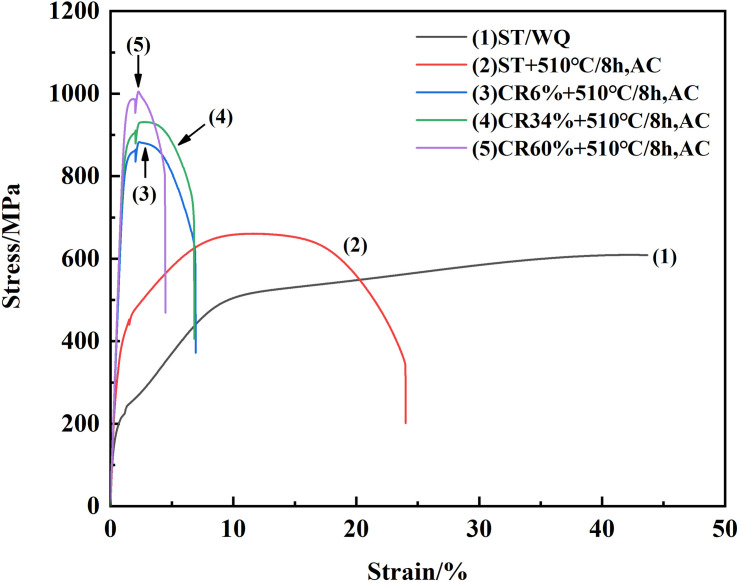
Tensile curves for the solution-treated specimen and TLM alloy plate subjected to cold rolling at the reductions of 0, 6, 34, 40, and 60% plus aging at 510°C for 8 h.

The tensile curves for TLM plate ST at 805°C for 1 h followed by water quenching and cold rolled with various reductions in conjunction with aging treatment at 510°C for 8 h are displayed in Figure 8. For the β solution-treated (805°C/1 h, WQ) condition, the curve demonstrates a so-called “double yielding” effect during the tensile deformation. The reason for this phenomenon is that when the temperature is selected in the beta phase region, the beta phase is in a relatively metastable condition, and the precipitation of martensite phase (α”) is prone to be promoted. The stress–strain curve with a fairly low value of apparent yielding stress is caused by the nucleation of the martensite phase and inheritance of room temperature deformation modes. Meanwhile, the emergence of the second yield point is attributed to the applied stress needed for the activation of certain slip systems, which could suppress the movement of the martensite laths during deformation. The tensile strength and elongation of the TLM alloy ST at 805°C for 1 h followed by water cooling are 609 MPa and 43.7%, respectively. Moreover, the tensile strength gradually increases with the increment in the reductions, while the elongation decreases with the increment in the reduction of cold rolling. The maximum value of tensile strength is as high as 1,005 MPa at cold rolling with a 60% reduction followed by aging treatment. However, the elongation of the TLM alloy plate is only about 4.5% in this condition. The TLM alloy plate has not visibly demonstrated the characteristic of plastic deformation. It can be inferred that the tensile strength and elongation of the TLM alloy plate are obviously different because of the volume percentage, size, and shape of α phases formed after cold deformation followed by aging treatment. The TLM alloy having finer α precipitations formed, based on the assisted nucleation and growth of dislocation tangles, or pile up during cold deformation with a reduction of 34%, possesses a higher tensile strength level. The main reason for this is considered to be the following factors including the geometrical size and volume percentage of α phase as well as the quantity and density of α phase interfaces, which are significantly influenced by the degree of cold-rolling deformation. Based on the precipitation strengthening criterion, the α precipitated phase refinement contributes to the enhancement of tensile strength for the TLM alloy (Gao et al., 2019; Bermingham et al., 2020). Furthermore, it can be noted that the room temperature elongation of the alloy plate subjected to solution annealing plus direct aging treatment is approximately 24%, and the tensile strength is about 660 MPa. The reason is that the formation of α precipitation phases with thick lamellar is suppressed owing to the lack of newly formed interfaces within the (ST + AG) sample, while they will be precipitated within the alloy plates after cold rolling in conjunction with aging treatment. These abundant newly formed α interfaces will hinder the dislocation slip or movement and result in the decrease in elongation at room temperature. Moreover, the tensile strength and elongation for the TLM alloy plate subjected to cold rolling at a reduction of 6% followed by 510°C aging treatment for 8 h are 882 MPa and 6.5%, respectively.

**FIGURE 9:**
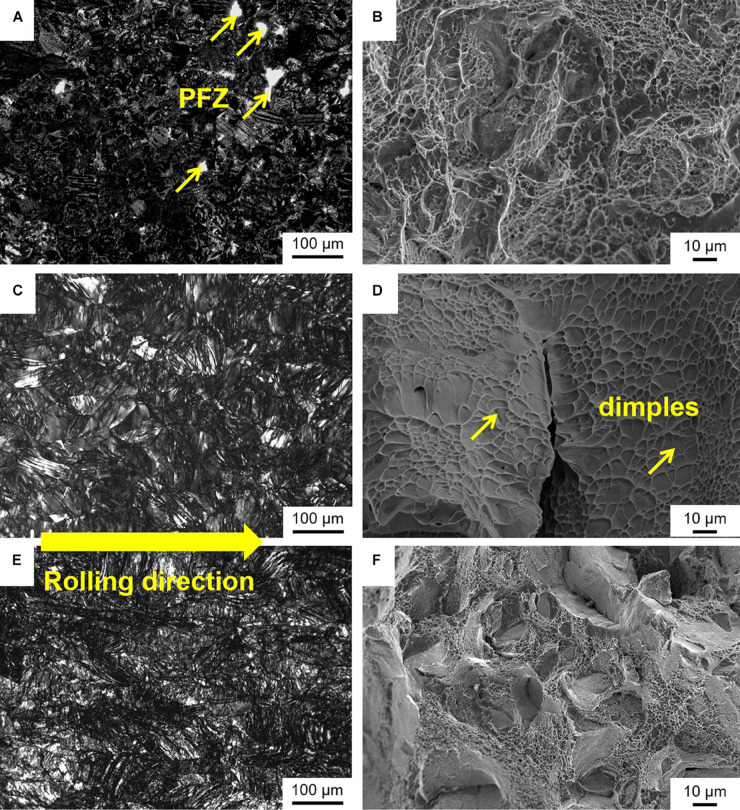
Microstructures and fracture surfaces for the TLM alloy plates subjected to cold rolling at various reductions followed by aging treatment at 510°C for 8 h: **(A,B)** 0% reduction. **(C,D)** 34% reduction; **(E,F)** 60% reduction.

The OM of the TLM alloy plates subjected to cold rolling with a reduction of 0% (solution treated), 34%, and 60% followed by 510°C aging treatment for 8 h and their corresponding fracture surfaces after tensile deformation are presented in Figure 9. In general, the OM and fracture surfaces are often used to analyze and characterize the details of microstructural evolution for the experimental alloy plates according to the morphology features and deformation behaviors to be predicted. Figures 9A,B indicate that α phases are precipitated in the β matrix, and a very small amount of micro precipitate-free zones (PFZ) can be observed in the TLM alloy plate subjected to solution annealing plus direct aging treatment. Interestingly, from Figures 9C,D, it can be observed that more and more α precipitations with smaller size form in the TLM alloy plate subjected to cold rolling at a reduction of 34% and followed by aging treatment. It is believed that the cold rolling plays a significant role in the uniform distribution and refinement of α precipitations and increasing in the number of secondary phases. As for the TLM alloy plate subjected to cold rolling at a reduction of 34%, many crystal defects induced by plastic deformation, such as dislocation tangles or pile up, contribute to provide more nucleation sites and suppress the over quick coarsening and growth of secondary phases during aging. Furthermore, it can be obviously observed in Figure 9D that the intergranular fracture mode is dominant in the TLM alloy plate subjected to cold rolling at a reduction of 34% followed by aging treatment. As seen from the details of fracture surface morphology, the alloy plate shows ductile fracture characteristic to some extent. The fracture surface is composed of massive dimples with a size of approximately 5–11 μm. This fracture mode is associated with the more uniform and smaller secondary phases precipitated in the β grains and the acceptable ductility with 6.9% at room temperature. The finer scale of secondary phases precipitated in the process of aging treatment after cold rolling will result in the formation of plenty of interfaces between alpha and beta phases. These interfaces can be considered as a lot of effective obstacles to dislocation movement and cause visible increase in the tensile strength for the TLM and other titanium alloys (Cai et al., 2013; Ozan et al., 2019). The OM and fracture surface (SEM image) for the TLM alloy plate subjected to cold rolling at a reduction of 60% and followed by aging treatment are presented in Figures 9E,F. The fracture surface shows the evidence of brittle failure to some extent due to the formation of a thick lath-like α phase in the grain boundary, which could result in a decrease in elongation after tensile deformation at room temperature.

## Conclusion

The microstructure characteristics of the TLM alloy plates cold deformed at various reductions and its influence on precipitated phases during aging treatment were mainly studied in this work. The TLM alloy plates possessed various mechanical properties owing to the different morphology, size, and volume fraction of α precipitated phases after cold-rolling deformation with various reductions (0, 6, 34, 40, and 60%) in conjunction with aging treatment at 510°C for 8 h. These main conclusions could be summarized from this work:

(1) The <110> texture was prone to form in the β solution-treated TLM alloy plates subjected to cold-rolling deformation with various reductions at room temperature. Dislocation tangles were visibly observed in the β matrix after cold rolling.
(2) The α precipitated phases formed after cold rolling with various reductions (0, 34, and 60%) in conjunction with aging treatment possessed various characteristics. The dimension of α precipitated phases in the alloy plate subjected to cold rolling was smaller than that of the α phases transformed after a direct solution treatment in conjunction with aging.
(3) When the alloy plate was subjected to cold rolling at a reduction of 60% in conjunction with aging, the maximum tensile strength could be achieved, while the elongation was relatively low. A large number of newly generated subgrain boundaries and interfaces were prone to be formed after cold deformation with various reductions at room temperature. The precipitated phases with thick lamellar were formed, which could be considered as one of the principal reasons for the relatively low elongation.
(4) When the alloy plate was subjected to cold rolling at a reduction of 34% in conjunction with aging, the smaller α precipitated phases could be formed within the β matrix, resulting in the relatively high tensile strength of 931 MPa and the acceptable elongation of 6.9%. Therefore, the TLM alloy will be deemed as a potential material in the orthopedic field.

## Data Availability Statement

The raw data supporting the conclusions of this article will be made available by the authors, without undue reservation.

## Author Contributions

JC took charge of the manuscript writing and data analysis. JL took charge of the technical guidance and supervision. SY took charge of literature research and providing research idea. ZD and XZ took charge of the microstructure characterization. WZ took charge of the mechanical properties testing. JG and HW took charge of the preparation of specimens. HS took charge of the cold rolling of alloy plates. ZY took charge of the texture analysis. All authors contributed to the article and approved the submitted version.

## Conflict of Interest

The authors declare that the research was conducted in the absence of any commercial or financial relationships that could be construed as a potential conflict of interest.
